# High Protein Intake at Lunch Is Negatively Associated with Blood Pressure in Community-Dwelling Older Adults: A Cross-Sectional Study

**DOI:** 10.3390/nu15051251

**Published:** 2023-03-02

**Authors:** Hélio José Coelho-Júnior, Samuel da Silva Aguiar, Ivan de Oliveira Gonçalves, Riccardo Calvani, Matteo Tosato, Francesco Landi, Anna Picca, Emanuele Marzetti

**Affiliations:** 1 Department of Geriatrics and Orthopedics, Università Cattolica del Sacro Cuore, 00168 Rome, Italy; 2 Graduate Program in Physical Education, Federal University of Mato Grosso, Cuiabá 78060-900, Brazil; 3 Physical Education Department, University Center—704/904 Seps Eq 702/902, Brasília 70390-045, Brazil; 4 Department of Health, Piaget University, Suzano 08673-010, Brazil; 5 Fondazione Policlinico Universitario “A. Gemelli”, IRCCS, 00168 Rome, Italy; 6 Department of Medicine and Surgery, LUM University, 70100 Apulia, Italy

**Keywords:** nutrition, meals, hypertension, cardiovascular risk, elderly

## Abstract

Background: The present study was conducted to explore the association between protein intake across the main meals and hypertension (HTN)-related parameters in community-dwelling Brazilian older adults. Methods: Brazilian community-dwelling older adults were recruited in a senior center. Dietary habits were assessed through 24 h recall. Protein intake was classified as high and low according to median and recommended dietary allowance values. Absolute and body weight (BW)–adjusted protein consumption levels were quantified and analyzed according to ingestion across the main meals. Systolic (SBP) and diastolic blood pressure (DBP) were measured using an oscilometric monitor. Participants were categorized as hypertensive according to physician diagnosis or the detection of high SBP and/or DBP values. Results: One hundred ninety-seven older adults were enrolled in the present study. Protein intake at lunch was independently and negatively associated with SBP. Furthermore, a lower prevalence of HTN (diagnosed by a physician) was observed in participants with higher intakes of protein. These results remained significant after adjustment for many covariates. However, significance was lost when kilocalories and micronutrients were included in the model. Conclusions: Findings of the present study indicate that protein intake at lunch was independently and negatively associated with systolic BP in community-dwelling older adults.

## 1. Introduction

Hypertension (HTN) is a chronic condition characterized by sustained elevations in blood pressure (BP) levels [1]. The highest prevalence of HTN is found in older adults, with more than 70% of those aged 65+ years being affected worldwide [2]. These data deserve concern, given that HTN is a leading cause of negative outcomes, including cardiometabolic, cerebrovascular, and renal events, in addition to premature death [2].

Changes in lifestyle habits are a cornerstone in the prevention and treatment of HTN [3]. Significant reductions in BP are achieved after adoption of healthy nutritional habits, including specific diet patterns (e.g., Dietary Approaches to Stop Hypertension [DASH] diet) [4], low sodium [5], and high potassium [6].

An increasing number of studies have investigated the association between protein intake levels and HTN-related parameters. Large cohort studies and meta-analyses have provided encouraging results, showing that people with high protein intake display low BP values [7,8,9,10]. However, other studies have reported higher BP levels in those with greater protein ingestion [11] or no associations between protein intake and BP values [12,13,14]. Most investigations examined mixed samples of adults from different age groups, while studies exclusively based on old populations are scarce.

The effects of protein intake on cardiovascular health might be dependent on protein sources [12] and distribution across meals [15]. For instance, Berryman et al. [15] examined a large cohort of American adults and found that greater protein intake early in the day was inversely associated with hemodynamic parameters.

Based on these premises, the present study was conducted to explore the association between protein intake and HTN-related parameters in community-dwelling Brazilian older adults. We also examined the possible influence of protein distribution across main meals on BP parameters.

## 2. Materials and Methods

### 2.1. Study Design

This was a cross-sectional study that investigated the association between protein intake and HTN-related parameters in community-dwelling older adults. The study protocol was approved by the Research Ethics Committee of the University of Campinas (Campinas, Brazil). All study procedures were conducted in compliance with the Declaration of Helsinki and Resolution 196/96 of the National Health Council. Participants were thoroughly informed about the study procedures and objectives before they provided written consent. The manuscript was prepared in accordance with the STROBE statement [16].

### 2.2. Participants

Participants were recruited by convenience between January 2016 and December 2018 in a community senior center located in Brazil. The community senior center offers daily sessions for flexibility, aquatic and multicomponent physical exercises, dance classes, adapted sports, nursing and medical care, and cognitive stimulation therapy. Candidate participants were considered eligible if they were 60 years or older, lived in the community, and possessed sufficient physical and cognitive abilities to perform all assessments required by the protocol. Candidates were excluded if they were on hormone replacement therapy and/or psychotropic drugs.

### 2.3. Hemodynamic Parameters

The procedures for BP measurement were adapted from the VII Joint National Committee on Prevention, Detection, Evaluation, and Treatment of High BP (JNC7) [1]. BP was measured in the morning for approximately 80 s. Participants were not fasting when BP was measured. Measurements were obtained three times with 1 min rest intervals, on three different days. Mean values were used for the analysis. BP was measured after participants remained seated for 15 min in a quiet room with feet parallel at one shoulder width, both forearms and hands on the table, supinated hands, back against the chair, without moving or talking. An automatic, noninvasive, and valid [17] arterial BP monitor (Microlife-BP 3BT0A, Microlife, Widnau, Switzerland) was used to measure systolic BP (SBP), diastolic BP (DBP), and heart rate (HR). An appropriate cuff was selected after measuring the arm circumference of each participant (Sanny, São Paulo, Brazil) and was placed at approximately the midpoint of the upper left arm (heart level).

### 2.4. Dietary Assessment

Food intake was assessed using a 24 h recall report. This method uses an open-ended questionnaire to provide quantitative and subjective estimations of actual food consumption [18]. Trained investigators asked the participants to describe in detail all foods they consumed on a meal-by-meal basis, including snacks, during the previous 24 h period. Interviews occurred on Tuesdays, Wednesdays, Thursdays, and Fridays to avoid bias associated with weekends. Participants were asked to describe in detail the cooking methods (e.g., fried, grilled, roasted), amounts in portions, product brands, sauces, spices, and condiments consumed, and the use of dietary supplements. The amounts of beverages consumed were also recorded, and participants were asked to describe if and how beverages were sweetened. Two-dimensional aids (e.g., photographs), household utensils (e.g., standard measuring cups and spoons), and food models were used as memory aids to assess portion sizes. Diet composition was estimated using NutWin software, version 1.5 (Federal University of São Paulo, Brazil).

### 2.5. Anthropometric Measurements and Hypertension Prevalence

A weight scale with a stadiometer was used to measure body weight (BW) and height. The body mass index (BMI) was subsequently calculated as follows:

(a) body weight (kg)/ height (m²).

Information pertaining to the prevalence of HTN was collected by two researchers through self-report and careful review of medical charts kept by the community senior center. Medical charts were updated every six months by a local physician. The presence of HTN was determined according to (a) physician diagnosis, or (b) the detection of high SBP and/or DBP values according to JNC7 [1].

### 2.6. Statistical Analysis

The normal distribution of variables was ascertained via the Shapiro–Wilk test. Continuous variables are expressed as the mean ± standard deviation (SD) or absolute numbers (percentage). Pearson’s correlation analysis was used to explore the association between protein intake, BP, and HR. Associations with a *p*-value lower than 0.05 were included in the regression analyses. The final model was adjusted for age, sex, BMI, kilocalories, and micronutrients. To test associations between categorical data, a chi-squared test was conducted. Variables were dichotomized into “high” and “low” levels based on the following median values: SPB = 135 mmHg, DBP = 77 mmHg, HR = 77 bpm, protein intake = 96.6 g/day, BW-adjusted protein intake = 1.5 g/kg of BW/day, calcium = 832 mg/day, magnesium = 392.7 mg/day, potassium= 3606 mg/day, and sodium = 1540 mg/day. BW-adjusted protein intake was also categorized as “high” versus “low” according to the recommended dietary allowance (RDA) for protein (0.8 g/kg of BW/day) and median values. Significance was set at 5% (*p*-value < 0.05) for all tests. Regression analyses were significant if the 95% confidence interval (CI 95%) did not include the value of 1. All analyses were performed using SPSS software (version 23.0, SPSS Inc., Chicago, IL, USA).

## 3. Results

### 3.1. Characterstics of Participants

One hundred ninety-seven older adults were enrolled in the present study. The main characteristics of participants are shown in Table 1. Participants were young older adults (mean age: 68.3 ± 6.8 years) and mostly female (83%). Mean BMI values (28.7 ± 5.0 kg/m^2^) indicated that participants were frequently overweight. HTN, based on a physician’s diagnosis, was highly prevalent in the study population (58.5%). A higher prevalence (64.6%) was observed according to high BP values. Mean SBP (134.3 ± 17.9 mmHg) was slightly above the cutoff values for HTN, whereas mean DBP (76.0 ± 10.8 mmHg) was marginally below [1]. Mean protein intake was noticeably higher than RDA values (1.5 ± 0.6 g per kg of BW) [19].

### 3.2. Associations between Dietary Habits and Hypertension-Related Parameters Using Continuous Data

Pearson’s correlations are shown in Table 2, Table S1, and Table S2. No significant associations were observed between SPB, DBP, and HR with absolute or BW-adjusted protein intake, or BW-adjusted protein consumption at breakfast and dinner. However, a significant association was observed between BW-adjusted protein consumption at lunch and SBP (r = −0.20, *p*-value = 0.007). Results did not change after adjustment for age, BMI, kilocalories, and micronutrient intake (r = −0.20, *p* = 0.009).

Results of linear regression are shown in Table 3. Unadjusted analyses indicated that absolute and BW-adjusted protein intake were significantly associated with SBP. A significant association was also observed between BW-adjusted protein consumption at lunch and SBP. Statistical significance of the association of absolute and BW-adjusted protein intake with SBP was lost when the model was adjusted for age, BMI, sex, kilocalories, and micronutrient intake. The association between BW-adjusted protein consumption at lunch and SBP remained significant.

### 3.3. Associations between Dietary Habits and Hypertension-Related Parameters Using Binary Data

Results of chi-squared statistics are shown in Table 4. A significant association was observed between HTN diagnosed by a physician and absolute and BW-adjusted protein intake. No significant associations were observed between protein consumption and HTN estimated based on BP values.

Binary regression was conducted using HTN diagnosed by a physician as a dependent variable (Table 5). In the unadjusted analysis, absolute and BW-adjusted protein intake were inversely associated with HTN. Results remained significant for absolute, but not BW-adjusted protein, when the model was adjusted for age, BMI, and sex. However, no significance was found when kilocalories and micronutrients were included as covariates in the analysis.

## 4. Discussion

The main findings of the present study indicate that protein intake at lunch was independently and negatively associated with SBP in a sample of community-dwelling older adults. Furthermore, a lower prevalence of HTN (diagnosed by a physician) was observed in participants with higher intakes of protein. These results remained significant after adjustment for age, BMI, and sex. Significance was lost when kilocalories and micronutrients were included in the model.

Numerous studies have examined the association between daily consumption of proteins, BP measures, and HTN. Investigations have produced conflicting results, with studies reporting positive, negative, and null relationships. In line with our findings, Der Kuil et al. [12] observed no significant relationships between total protein intake and changes in SBP and DBP in Dutch adults. The authors also noted that protein intake had no influence on the incidence of HTN [20]. Similar findings were reported by Tielemans et al. [13] in older Dutch men. Liu et al. [14] found no significant associations between protein intake and the prevalence of HTN in poorly nourished rural Chinese people. Increased protein intake also failed to ameliorate hemodynamic parameters in randomized clinical trials. Indeed, Hodgson et al. [21] did not report differences in BP values in Australian older adults who consumed protein supplements for two years.

Results of the INTERSALT study, which included more than 10,000 adults, indicated that dietary protein markers, urinary nitrogen, and urea excretion were negatively associated with SBP and DBP [7]. These findings were supported by secondary analyses of the Framingham cohort conducted in young and middle-aged adults [8,9]. In contrast, Umesawa et al. [22] observed that total protein intake was inversely associated with DBP in Japanese people. Regarding positive associations, Hajjar et al. [11] reported significant relationships between protein intake and SBP in North American adults.

These conflicting results might be explained by differences in sample characteristics (e.g., age, sex) [7], protein intake [23] and quality [13,22], HTN status [12], and the covariates included in the analyses [9,12,13,23]. Our sample was composed of overweight community-dwelling young older adults with a relatively high intake of proteins and controlled BP levels. In contrast, inverse associations seem to be stronger in older women [7] with untreated HTN [12]. An interesting scenario was recently offered by the study of He et al. [23], in which the relationship between protein consumption and HTN was U-shaped, suggesting that a moderate intake of protein had no influence on HTN. However, this view was not supported by Mehrabani [24], who noted a dose–response relationship between protein intake and BP values.

We observed that protein intake at lunch was independently and negatively associated with SBP. Another study indicated that consuming more protein early in the day was associated with a better cardiometabolic profile, including low BP and LDL cholesterol levels, whereas people who consumed more protein at dinner were more likely to display insulin resistance [15]. A possible explanation for these results is based on the role of protein consumption on satiety, and its consequences on calorie ingestion and circadian rhythm.

Protein consumption induces satiety [25]. In fact, people who consume protein-rich meals in the morning report more satiety over the day, whereas those who consume low-protein commonly experience hunger before and after the main meals [26]. Hunger is a major regulator of eating patterns [27,28]. Appetite involves subsequent energy intake in the forms of large amounts of food during discrete intervals, small amounts of food continuously over long periods, or snacks [27,28]. Such a variation in meal patterns changes hormonal synthesis [29,30] and is associated with weight gain [31]. In contrast, high protein intake in the morning is accompanied by a high expression of genes associated with lipid metabolism [32], as well as reduced glucose levels, better insulin sensitivity, and lower SBP values [26].

Taken together, these observations suggest that the relationship between high protein intake at lunch and low SBP observed in the present study might occur as a result of the effects of protein on satiety.

The impact of protein on BP is also dependent on protein composition and amino acid (AA) availability [9,12,13,23,33]. For instance, L-arginine is an essential AA that serves as a substratum for nitric oxide production, which acts as a major endothelium vasodilator [34]. Randomized clinical trials have observed that chronic supplementation with L-arginine might reduce BP levels, regardless of HTN status [35,36]. Tryptophan is an aromatic AA precursor for the synthesis of serotonin [37]. When activated, serotonin receptors induce direct arterial vasoconstriction in different vascular beds [38]. Serotonin receptors are also expected to be hyperactivated in HTN [38]. Tyrosine is a precursor for norepinephrine synthesis, consequently affecting sympathetic activity [37]. Peripheral and central administration of tyrosine reduces BP in normotensive and hypertensive rats in a dose-dependent fashion [39,40]. In humans, the intake of tyrosine was negatively associated with BP in people from the Rotterdam study cohort [41]. However, no longitudinal associations were observed between AA intake and HTN incidence [41].

Other authors have argued that studying the effects of many AAs simultaneously might provide a more realistic scenario than investigating their individual effects [42]. Using a principal component analysis approach, Teymoori et al. [42] noted that the consumption of branched, alcoholic, and aromatic A As was associated with an increased risk of HTN, whereas sulfuric and small AAs showed a trend to be associated with lower HTN incidence. Hence, although there are many AA candidates, studies are required to provide a more detailed picture.

A complementary explanation for our results involves the association between muscle mass and hemodynamic parameters. Protein intake is a major regulator of muscle mass by providing essential AAs, mainly branched-chain AAs, to stimulate muscle protein synthesis [43,44]. The failure to properly promote muscle anabolism predisposes one to the gradual loss of muscle mass [45], a scenario called muscle atrophy, and raises the risk of developing numerous health-related conditions, including frailty and sarcopenia [46,47].

More recently, an increasing number of studies have found that community-dwelling adults with low muscle mass markers display elevated BP and a high prevalence of HTN [48,49]. The exact mechanisms underlying this scenario still need to be elucidated but might include arterial stiffness, oxidative stress, and inflammation [50,51].

Our study is not free of limitations. First, some investigations observed significant associations between protein sources—animal and vegetal—and BP-related parameters. Specifically, He et al. [23] found a U-shaped relationship between plant-based protein and the incidence of HTN, whereas the association with animal protein, mainly white and red meat, was J-shaped. In addition, protein sources might influence cardiometabolic risk factors [8]. Although findings on the subject are conflicting [8,9,12,13,14,22,33], this topic deserves deeper exploration. Second, variables that might influence protein intake and/or BP, including physical activity and exercise, oral health, medication use, and the prevalence of chronic conditions, were not controlled for. Third, although BP was measured using valid oscilometric BP monitors, ambulatorial BP monitoring seems to be a better predictor of cardiovascular events [52]. Fourth, our findings were obtained in community-dwelling Brazilian older adults, and extrapolations to other countries or in other settings should be made with caution. Fifth, other instruments than the 24 h dietary recall might be necessary to capture dietary patterns over long periods (e.g., diet diary). Sixth, participants of the present study had a high mean BW-adjusted protein intake (1.5 ± 0.6 g/kg of BW). A recent systematic review found that this scenario is commonly observed in studies investigating protein intake in older adults [53]. Therefore, the possibility that findings might be different in people with protein intake levels near or below the RDA cannot be ruled out. Finally, the cross-sectional design of the study does not allow any inference to be drawn on the time course of changes in the variables considered or on cause–effect relationships.

## 5. Conclusions

Findings of the present study indicate that protein intake at lunch was independently and negatively associated with systolic BP in community-dwelling older adults.

## Figures and Tables

**Table 1:** Characteristics of study participants (n = 197).

Variables	Mean or Absolute Value	SD or %
Age, years	68.3	6.8
Weight, kg	68.9	12.0
Height, m	154.7	8.1
BMI, kg/m²	28.7	5.0
Female	164	83.0
Energy intake, kcal	1846.1	545.1
Protein, g	106.1	40.9
Protein, g/kg	1.5	0.6
Protein at breakfast, g/kg	0.18	0.11
Protein at lunch, g/kg	0.83	0.42
Protein at dinner, g/kg	0.35	0.41
Calcium, mg	817.8	382.5
Magnesium, mg	405.6	123.7
Sodium, mg	1625.7	786.3
Potassium, mg	3680.1	1148.3
SBP, mmHg	134.3	17.9
DBP, mmHg	76.0	10.8
HR, bpm	77.3	11.3
HTN, diagnosed by a physician	115	58.5
HTN, diagnosed according to the JNC7	127	64.6

Continuous data are shown as data ± standard deviation. The prevalence of female participants and hypertension are shown as absolute and relative values. SBP = systolic blood pressure; DBP = diastolic blood pressure; HR = heart rate; BMI = body mass index; HTN = hypertension; SD = standard deviation.

**Table 2:** Unadjusted Pearson’s correlation (n = 197).

	Protein, g	Protein, g/kg	Protein at Breakfast, g/kg	Protein at Lunch, g/kg	Protein at Dinner, g/kg
SBP, mmHg	−0.11, 0.136	−0.14, 0.061	−0.005, 0.944	**−0.20, 0.007**	−0.01, 0.837
DBP, mmHg	−0.03, 0.673	−0.05, 0.449	−0.01, 0.861	−0.03, 0.667	−0.08, 0.277
HR, bpm	0.02, 0.770	−0.01, 0.839	−0.12, 0.094	−0.07, 0.317	0.02, 0.728

Data are presented as Pearson’s correlation (r), *p*-value. SBP = systolic blood pressure; DBP = diastolic blood pressure; HR = heart rate; bold denotes *p* < 0.05.

**Table 3:** Linear regression for protein intake and systolic blood pressure.

	Univariate β (95% CI)	Adjusted β (95% CI)
Absolute protein intake	−0.047 (−0.109, 0.015)	−0.086 (−0.181, 0.009)
BW-adjusted protein intake	−3.671 (−7.519, 0.178)	−5.580 (−11.682, 0.522)
BW-adjusted protein intake at lunch	**−7.995 (−13.751, −2.238)**	**−9.399 (−15.997, −2.802)**

Adjusted for age, sex, and body mass index; kilocalories, calcium, potassium, sodium, and magnesium; HTN = hypertension; CI = confidence interval; bold denotes *p* < 0.05.

**Table 4:** Frequency (%) of the distribution of older adults in hypertension-related parameters.

Variables	HTN	HTN *	SBP	DBP	SBP *	DBP *	HR
Protein, g							
<93.6	**64 (32.5%)**	21 (12.0%)	39 (22.2%)	39 (22.2%)	47 (26.7%)	32 (18.2%)	39 (22.7%)
≥93.6	**51 (25.9%)**	40 (22.9%)	51 (29.0%)	53 (30.1%)	70 (39.8%)	46 (26.1%)	50 (29.1%)
Protein, g/kg body weight							
<0.8	9 (4.5%)	2 (1.1%)	6 (3.4%)	3 (1.7%)	7 (3.9%)	2 (1.1%)	6 (3.4%)
≥0.8	108 (54.0%)	61 (34.3%)	86 (48.0%)	91 (50.8%)	112 (62.6%)	78 (43.6%)	85 (48.6%)
Protein, g/kg body weight							
<1.0	27 (13.5%)	10 (5.6%)	18 (10.1%)	18 (10.1%)	23 (12.8%)	15 (8.4%)	16 (9.1%)
≥1.0	90 (45.0%)	53 (29.8%)	74 (41.3%)	76 (42.5%)	96 (53.6%)	65 (36.3%)	75 (42.9%)
Protein, g/kg body weight							
<1.5	**65 (33.0%)**	27 (15.4%)	44 (25.0%)	42 (23.9%)	58 (33.0%)	35 (19.9%)	42 (24.4%)
≥1.5	**50 (25.4%)**	34 (19.4%)	46 (26.1%)	50 (28.4%)	59 (33.5%)	43 (24.4%)	47 (27.3%)

HTN = hypertension, SBP = systolic blood pressure; DBP = diastolic blood pressure; HR = heart rate, * according to the JNC7.

**Table 5:** Binary regression for protein intake and hypertension.

	Unadjusted OR (95% CI)	Adjusted OR (95% CI) *
Absolute protein intake	**0.537 (0.302, 0.953)**	0.569 (0.280, 1.156)
Body weight-adjusted protein intake	0.550 (0.301, 1007)	0.676 (0.338, 1.352)

* Adjusted for age, sex, and body mass index; kilocalories, calcium, potassium, sodium, and magnesium; bold denotes *p* < 0.05; OR = odds ratio; HTN = hypertension; CI = confidence interval.

## Data Availability

Data are available at reasonable request from corresponding authors.

## Supplementary Materials

The following supporting information can be downloaded at: https://www.mdpi.com/article/10.3390/nu15051251/s1, Table S1: Adjusted Pearson’s correlation (n = 197); Table S2: Adjusted Pearson’s correlation (n = 197).
