# Supplementary material for: High Protein Intake at Lunch Is Negatively Associated with Blood Pressure in Community-Dwelling Older Adults: A Cross-Sectional Study
Source: Nutrients. 2023 Mar 2;15(5):1251. doi: 10.3390/nu15051251 (PMC10005279; doi:10.3390/nu15051251)
Supplement: Supplementary file 1 [file nutrients-15-01251-s001.zip › nutrients-2229943-supplementary.pdf]

Table S1. Adjusted Pearson's correlation (n=197).

	Protein, g	Protein, g/kg	Protein at breakfast, g/kg	Protein at lunch, g/kg	Protein at dinner, g/kg
SBP, mmHg	-0.11, 0.152	-0.09, 0.203	0.05, 0.574	**-0.19, 0.021**	0.06, 0.552
DBP, mmHg	0.07, 0.351	0.08, 0.256	0.03, 0.976	0.03, 0.940	0.01, 0.592
HR, bpm	-0.00, 0.952	0.00, 0.953	-0.14, 0.082	-0.05, 0.541	0.02, 0.718

Data are presented as Pearson's correlation (r), P-value. SBP= Systolic blood pressure; DBP= Diastolic blood pressure; HR= Heart rate; *Adjusted for age, sex, and body mass index; Bold= P<0.05.

Table S2. Adjusted Pearson's correlation (n=197).

	Protein, g	Protein, g/kg	Protein at breakfast, g/kg	Protein at lunch, g/kg	Protein at dinner, g/kg
SBP, mmHg	-0.14, 0.152	-0.14, 0.203	0.08, 0.291	**-0.20, 0.009**	0.00, 0.933
DBP, mmHg	0.07, 0.351	0.09, 0.256	0.07, 0.369	0.02, 0.627	-0.00, 0.897
HR, bpm	0.04, 0.952	0.06, 0.953	-0.08, 0.270	-0.03, 0.557	0.04, 0.550

Data are presented as Pearson's correlation (r), P-value. SBP= Systolic blood pressure; DBP= Diastolic blood pressure; HR= Heart rate; *Adjusted for age, sex, and body mass index, kilocalories, calcium, potassium, sodium, and magnesium; Bold= P<0.05.
